# Pentobarbital Anesthesia Suppresses the Glucose Response to Acute Intermittent Hypoxia in Rat

**DOI:** 10.3389/fphys.2021.645392

**Published:** 2021-03-05

**Authors:** Polina E. Nedoboy, Callum B. Houlahan, Melissa M. J. Farnham

**Affiliations:** ^1^Cardiovascular Neuroscience Unit, Heart Research Institute, Newtown, NSW, Australia; ^2^Department of Physiology, School of Medical Sciences, Faculty of Medicine and Health, The University of Sydney, Camperdown, NSW, Australia

**Keywords:** anesthesia-general, acute intermittent hypoxia, Sprague Dawley rat, blood glucose, glucoregulatory circuit, pentobarbital

## Abstract

A key feature of sleep disordered breathing syndromes, such as obstructive sleep apnea is intermittent hypoxia. Intermittent hypoxia is well accepted to drive the sympathoexcitation that is frequently associated with hypertension and diabetes, with measurable effects after just 1 h. The aim of this study was to directly measure the glucose response to 1 h of acute intermittent hypoxia in pentobarbital anesthetized rats, compared to conscious rats. However, we found that while a glucose response is measurable in conscious rats exposed to intermittent hypoxia, it is suppressed in anesthetized rats. Intermittent hypoxia for 1, 2, or 8 h increased blood glucose by 0.7 ± 0.1 mmol/L in conscious rats but had no effect in anesthetized rats (−0.1 ± 0.2 mmol/L). These results were independent of the frequency of the hypoxia challenges, fasting state, vagotomy, or paralytic agents. A supraphysiological challenge of 3 min of hypoxia was able to induce a glycemic response indicating that the reflex response is not abolished under pentobarbital anesthesia. We conclude that pentobarbital anesthesia is unsuitable for investigations into glycemic response pathways in response to intermittent hypoxia in rats.

## Introduction

Obstructive Sleep Apnea (OSA) (Heinzer et al., 2015; Yacoub et al., 2017) is a highly prevalent, but underdiagnosed condition characterized by repetitive airway collapse during sleep. OSA affects up to 30% of the population (Peppard et al., 2013) and is present in ∼70% of diabetics (Pamidi and Tasali, 2012; Rajan and Greenberg, 2015). Intermittent hypoxia (IHx), caused by repetitive collapse of the airways during OSA, is considered a key driver of insulin resistance and hence the development of type 2 diabetes. Current therapies prevent the physical collapse of the airways but are hindered by poor compliance and conflicting reports of improved cardio-metabolic health (da Silva Paulitsch and Zhang, 2019). Although OSA is commonly linked to obesity, of growing concern is the prevalence (25–30%) of OSA in healthy, non-overweight people (Pamidi et al., 2012; Gray et al., 2017), who, without any other risk factors, show signs of pre-diabetes. The natural history of cardio-metabolic impairment in the context of OSA and obesity is unclear, therefore causal directions remain elusive. Activation of the sympathetic nervous system (Leung et al., 2012) is widely recognized as an important mediator of OSA-induced pathophysiology, although mechanisms remain unproven.

IHx models developed in cell culture (Hunyor and Cook, 2018) and rodents (Polak et al., 2013; Rafacho et al., 2013; Farnham et al., 2019) are therefore used to simplify the disease process and isolate the hypoxia driven effects. The cardio-metabolic effects of IHx are rapid with elevated sympathetic activity (Farnham et al., 2019) and glucose after 1–2 h (Rafacho et al., 2013) in rats, and elevated glucose after 3 h (Newhouse et al., 2017) in humans. The sympathetic effects of OSA are well accepted, with patients presenting with increased daytime muscle sympathetic activity (Narkiewicz and Somers, 1997). IHx in *anesthetized* rodent models (Dick et al., 2007; Xing and Pilowsky, 2010; Blackburn et al., 2018; Kakall et al., 2018b; Kim et al., 2018; Roy et al., 2018; Farnham et al., 2019) and *conscious* humans (Louis and Punjabi, 2009; Gilmartin et al., 2010; Tamisier et al., 2011) also both demonstrate persistent increases in sympathetic nerve activity. In the case of chronic IHx, *conscious* rodent models develop increases in blood pressure (Sharpe et al., 2013) and glucose dysregulation (Polak et al., 2013; Fu et al., 2015). Rafacho et al. (2013) was the first to demonstrate that just 1 h of acute IHx elevated blood glucose in *conscious* rats which was attributed to sympathoactivation since administration of a β-blocker prevented this response (Rafacho et al., 2013). However in mice, β-adrenoceptor blockade had no effect, but α-adrenoceptor blockade and adrenomedullectomy blocked the response (Jun et al., 2014). While the mechanism remains contentious, these findings are both supportive of sympathoactivation driving the glucose response to acute IHx. However, no one has directly measured sympathetic activity and blood glucose in response to acute IHx. The purpose of this study was to measure the blood glucose response to 1 h of acute IHx in anesthetized rats and compare with conscious rats.

Here we report that pentobarbital anesthesia suppresses the increase in blood glucose seen in conscious rats. We report that increases in blood glucose are measurable after 1 and 2 h of IHx in conscious rats but are absent in anesthetized rats subjected to either 10 or 16 episodes of IHx within 1 h. We modified multiple experimental parameters including fasting, vagotomy, use of paralytic agents and “priming” with 1 h of conscious IHx before anesthesia. There was a significant but biologically irrelevant effect of “priming” leading to the conclusion that pentobarbital anesthesia is incompatible with investigations of IHx-induced changes in blood glucose.

## Materials and Methods

### Animals

Procedures and protocols were approved by the Sydney Local Area Health District Animal Care and Ethics Committee and conducted in accordance with the Australian codes of practice for the care and use of animals for scientific purposes. Rats are used as the experiments described involve an integrative approach and no artificial models of these systems currently exist.

Experiments were conducted on *n* = 78 adult male Sprague-Dawley (SD) rats (300–500 g; Animal Resource Centre, Perth, Australia).

Animals were housed in 12 h light cycle with lights “on” from 7 a.m. to 7 p.m. This constitutes the rat’s “night” when they spent most their time sleeping. We conducted all our experiments during the “night” cycle to align with the human condition of OSA.

### Measurement of Blood Glucose

Great care was taken to ensure minimal stress during the blood glucose measurement procedure in conscious untrained animals. Rats (either unfasted or 3 h fasted) were either allowed to walk freely into a dark cloth “sock” or in most cases remained in their home cage without any form of restraint. A scalpel was used to make a small nick at the tip of the tail. The first drop of blood was discarded, and the second drop was drawn up into a glucose test strip attached to a glucometer (AccuCheck or LifeSmart).

Immediately after the conclusion of the IHx or Sham protocol, the tail nick was reopened with gentle abrasion, while the rat remained unrestrained. The first drop of blood was discarded and the second used in the glucometer.

In the anesthetized animals, the tail nick and blood collection were conducted in the same manner.

### Intermittent Hypoxia

#### Conscious

Following blood glucose measurement, a single rat was placed in a small plastic container for 10 min before the IHx protocol was commenced. Rats that underwent the 8 h protocol were housed in groups of 3 and the experiment conducted in their home cage. Oxygen levels within the hypoxia chamber were continuously monitored with an OxyStar (CWE). A customized GSM-3 (CWE) programmable gas mixer was used to deliver 4 different gas mixes at 4 different flow rates to rapidly cycle between 21% O_2_ and 6/10% O_2_. In the conscious cohort of animals, 4 different IHx protocols were used, with 3 corresponding Sham protocols which consisted of normal room air (21% O_2_) being delivered to the animal at the same flow rates and timing as the IHx protocol:

1. 1 h (10 episodes) of 1 min of 10 ± 1% O_2_ in N_2_, each separated by a 5 min recovery period of 21% O_2_ (*n* = 9); Sham (*n* = 8).
2. 1 h (16 episodes) of 1 min of 6 ± 0.5% O_2_ in N_2_ each separated by a 2.5 min recovery period of 21% O_2_ (*n* = 7). The animals in this group were then anesthetized and surgically prepared for the anesthetized protocol and referred to as the “primed” group.
3. 2 h (16 episodes/h) of 1 min of 6 ± 0.5% O_2_ in N_2_ each separated by a 2.5 min recovery period of 21% O_2_ (n = 8); Sham (*n* = 6).
4. 8 h (16 episodes/h) of 1 min of 6 ± 0.5% O_2_ in N_2_ each separated by a 2.5 min recovery period of 21% O_2_ (*n* = 9); Sham (*n* = 9).

Immediately after the conclusion of the IHx or Sham protocol, blood glucose was measured again.

#### Anesthetized

Following baseline blood glucose measurements and blood gas analysis to ensure the animals were in good metabolic health, the IHx protocol (*n* = 26) was commenced and consisted of either:

5. 10 episodes of 45 s of 10% O_2_ in N_2_, each separated by a 5 min recovery period (Farnham et al., 2019) (*n* = 18).
6. 1 h (∼16 episodes) of 45 s of 10% O_2_ in N_2_ each separated by 3min recovery period (*n* = 8).

The Sham protocol (*n* = 7) consisted of the same time frames, but without any alteration of oxygen content of the inspired air.

### Single 3 min Hypoxia Challenge

In a subset of 2 anesthetized animals, 2 separate 3 min challenge of 10% O_2_ were conducted 30 min apart and 60 min following the conclusion of IHx. Blood glucose was measured just prior to the 3 min challenge and immediately after.

### Surgical Preparation

*N* = 33 rats were initially anesthetized with an intraperitoneal injection of pentobarbital sodium (65 mg/kg; Lethobarb). All animals were placed on a homeothermic heat mat to maintain core body temperature at 37 ± 0.5°C. The right carotid artery was cannulated for the measurement of arterial blood pressure and the right jugular vein was cannulated for the administration of fluids and drugs. A continuous infusion of pentobarbital in saline was commenced to deliver 65 mg/kg at a rate of 2 ml/h. Anesthetic depth was monitored continuously and anesthetic delivery was adjusted as necessary. A tracheostomy was performed to permit mechanical ventilation with room air supplemented with 100% O_2_. *N* = 4 did not have supplemental O_2_. Most animals (*n* = 21) were bilaterally vagotomized before being mechanically ventilated and paralyzed with pancuronium bromide (0.8 mg/kg i.v., followed by an infusion of 0.8 mg/kg/h of pancuronium in 0.9% saline at a rate of 2 ml/h; Astra Zeneca, Australia), while n = 4 were not vagotomized but mechanically ventilated and paralyzed with pancuronium bromide. In other instances (*n* = 9 the vagii were left intact and the animals entrained to the ventilator. The IHx or Sham protocol started within 1–1.5 h from the induction of anesthesia. Prior to the commencement of the IHx or Sham protocol, 0.2 ml of arterial blood was withdrawn for respiratory and electrolyte blood gas analysis (VetStat; IDEXX Laboratories, United States). Ventilation was adjusted, if necessary, to keep blood gases within physiologically normal ranges. After the final blood glucose measurement, a final blood gas analysis was conducted to ensure readings were still within physiological range.

### Data Analysis

Recordings of arterial blood pressure, expired CO_2_, heart rate and core temperature in the anesthetized rats were acquired using a CED 1401 ADC system and Spike 2 acquisition and analysis software (v. 8.11b; Cambridge, United Kingdom). Recordings of the O_2_ levels within the conscious hypoxia chambers was also acquired with a CED 1401 ADC system. Blood glucose measurements from the glucometer were entered into an excel spreadsheet which was used to calculate the change in blood glucose following the IHx or Sham protocol. Statistical analysis was conducted in Graph Pad Prism software (v9). Non-parametric *t*-tests (Mann-Whitney) or one-way ANOVA (Kruskal-Wallis with *post hoc* Dunn’s multiple comparisons tests) were performed due to small sample sizes. Data are presented as mean ± SEM and *P* < 0.05 was deemed significant.

## Results

### 1, 2, and 8 h of Acute Intermittent Hypoxia Elevates Blood Glucose in Conscious Rats

**FIGURE 1:**
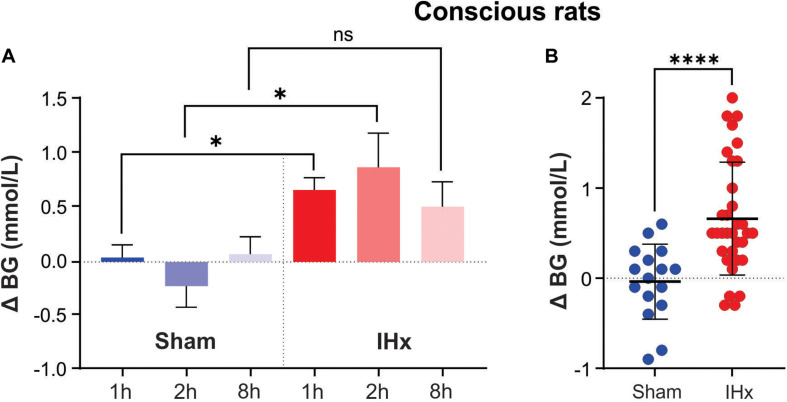
Intermittent hypoxia (IHx) elevates blood glucose in conscious rats. **(A)** The Sham condition of intermittent room air delivered at the same flow rate and volume as that of intermittent hypoxia (IHx) for 1, 2, or 8 h, does not elevate blood glucose in conscious rats. 1 and 2 h of IHx significantly elevated blood glucose compared to the respective Sham conditions. **(B)** Individual data points for Sham and IHx treatment groups. In both cases 1, 2, and 8 h are grouped together. ^∗^*P* < 0.05; ^****^P < 0.001. BG, blood glucose; Sham, intermittent room air; IHx, intermittent hypoxia.

1 h of 10% IHx and 1 h of 6% IHx raised blood glucose by 0.7 ± 0.4 vs. 0.6 ± 0.6 mmol/L, respectively (Figure 1A) and so the data were grouped together. In agreement with the findings of Rafacho et al. (2013), 1 h of IHx elevated blood glucose (0.7 ± 0.4 vs. 0.0 ± 0.3 mmol/L; *P* = 0.0023; Figure 1A), regardless of fasted state or severity/frequency of hypoxia challenges.

Sham for 1 h (0.0 ± 0.3 mmol/L), 2 h (−0.2 ± 0.5 mmol/L), or 8 h (0.1 ± 0.4 mmol/L) had no effect on blood glucose (*P* = 0.6696; Kruskal-Wallis; Figure 1A) indicating that the experimental conditions were not inducing a confounding stress response.

IHx for 2 h (0.9 ± 0.9 mmol/L) and 8 h (0.5 ± 0.6 mmol/L) both increased blood glucose to the same degree as 1 h IHx 0.7 ± 0.4 mmol/L; *P* = 0.3266; Kruskal-Wallis; Figure 1A), however only 1 and 2 h increases were significantly elevated compared with the equivalent Sham group. Figure 1B shows all data points grouped into either Sham or IHx groups. Raw blood glucose readings are presented in Table 1 for each of the groups.

**TABLE 1:** Baseline and final blood glucose readings.

Treatment group	*n*	Baseline BG mmol/L (mean ± SD)	Final BG mmol/L (mean ± SD)
**Conscious**			
Sham 1 h (all)	8	5.7 ± 0.8	5.7 ± 0.8
Sham 1 h (fasted)	2	4.5 ± 0.1	4.6 ± 0.1
Sham 1 h (non-fasted)	6	6.1 ± 0.4	6.1 ± 0.6
Sham 2 h (all fasted)	6	5.2 ± 0.6	5.0 ± 0.6
Sham 8 h (all fasted)	6	5.9 ± 0.6	6.0 ± 0.3
IHx 1 h 10% (all)	9	5.7 ± 1.2	6.4 ± 1.2
IHx 1 h 10% (fasted)	5	5.8 ± 1.1	6.6 ± 0.7
IHx 1 h 10% (non-fasted)	4	5.6 ± 1.5	6.1 ± 1.5
IHx 2 h 6% (all fasted)	8	6.2 ± 0.7	7.1 ± 1.3
IHx 8 h 6% (all fasted)	9	5.4 ± 0.4	5.9 ± 0.7
**Anesthetized**			
Sham (all)	7	5.3 ± 0.7	4.8 ± 0.6
Sham (vagotomized + paralyzed)	4	5.0 ± 0.8	4.5 ± 0.6
Sham (paralyzed)	3	5.6 ± 0.2	5.2 ± 0.5
IHx 1 h 10% (all)	15	4.9 ± 0.6	4.8 ± 0.8
IHx 1 h 10% (vagotomized + paralyzed)	11	5.0 ± 0.7	4.7 ± 0.9
IHx 1 h 10% (paralyzed)	4	4.7 ± 0.5	5.1 ± 0.7
Single 3 min Hx 10% (all vagotomized + paralyzed)	2	4.4 ± 0.9	6.6 ± 1.4
**Priming**			
Conscious IHx 1 h 6%	7	5.3 ± 0.4	5.9 ± 0.4
Anesthetized IHx 10% primed (all)	11	4.9 ± 0.4	5.2 ± 0.5
Anesthetized IHx 10% primed (vagotomized + paralyzed)	2	4.7 ± 0.2	4.6 ± 0.7
Anesthetized IHx 10% primed (non-vagotomized + non-paralyzed)	9	4.9 ± 0.4	5.3 ± 0.4

### 1 h of Acute Intermittent Hypoxia Fails to Elevate Blood Glucose in Anesthetized Rats

**FIGURE 2:**
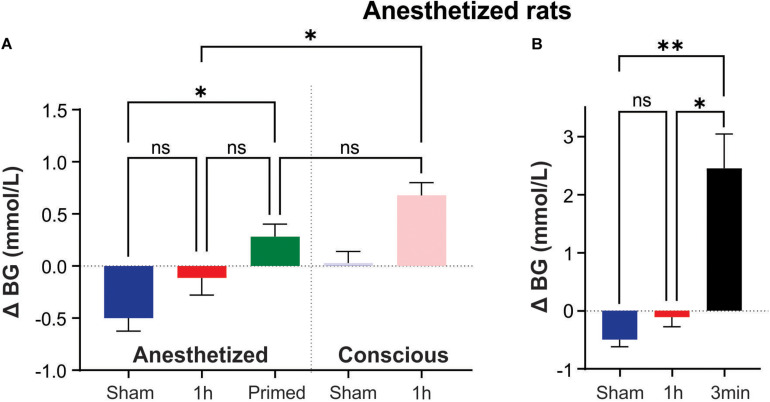
Intermittent hypoxia (IHx) does not elevate blood glucose in anesthetized rats. **(A)** Neither Sham, nor 1 h of IHx elevated blood glucose in pentobarbital anesthetized rats. Primed rats which received 1 h of conscious IHx prior to being anesthetized did have a significantly elevated blood glucose response to IHx under anesthesia when compared with Sham. This response was no different to the conscious Sham response and unlikely to be biologically meaningful. **(B)** A single 3 min hypoxia did evoke a large and significant increase in blood glucose compared to both Sham and 1 h IHx. ^∗^*P* < 0.05; ^∗∗^*P*, 0.001. BG, blood glucose; Sham, intermittent room air; Primed, 1 h conscious IHx prior to anesthetized IHx; 1 h, 1 h of IHx; 3 min, a single 3 min challenge of 10% oxygen.

Under pentobarbital anesthesia, blood glucose did not rise but appeared to decrease following 1 h of Sham (−0.5 ± 0.3 mmol/L; Figure 2A). 1 h of IHx produced the expected effects on cardiovascular parameters as described before Table 2 (Farnham et al., 2019) but failed to raise blood glucose (−0.1 ± 0.6 mmol/L; Figure 2A) and was no different to the change in blood glucose of the Sham group (*P* = 0.8787; Kruskal-Wallis). None of the protocol adjustments (O_2_ supplementation, vagotomy, paralytic agents or length of hypoxia challenge (45 vs. 60 s) had any effect so data are presented as a single group.

**TABLE 2:** Baseline and final blood pressure (BP), heart rate (HR) and end-tidal CO_2_ (ETCO_2_).

Treatment group	*n*	Baseline BP mmHg (mean ± SD)	Final BP mmHg (mean ± SD)	Baseline HR bpm (mean ± SD)	Final HR bpm (mean ± SD)	Baseline ETCO_2_ % (mean ± SD)	Final ETCO_2_ % (mean ± SD)
**Anesthetized**							
Sham	7	124.517.8	116.625.5	379.748.9	349.447.5	1.60.6	1.60.6
IHx 1 h 10%	15	110.718.3	10921.3	370.644.0	348.848.0	2.00.6	2.20.8
Single 3 min Hx 10%	4	81.815.9	86.55.2	312.122.9	302.217.5	3.10.1	3.20.1
**Priming**							
Anesthetized IHx 10% primed	10	119.613.4	115.717.3	357.834.2	325.726.3	2.31.1	2.31.1

However, a priming stimulus of 1 h IHx in conscious animals prior to the anesthetized IHx resulted in a significant rise in blood glucose compared with Sham anesthetized animals (0.3 ± 0.4 vs. −0.5 ± 0.3 mmol/L; *P* = 0.0094; Kruskal-Wallis; Figure 2A). While promising for conducting these experiments under anesthesia, this result is unlikely to be biologically relevant as the change in blood glucose was no different to that seen after 1 h of Sham treatment in conscious rats (*P* > 0.9999; Kruskal-Wallis).

A single 3min hypoxia challenge was able to evoke a significant elevation of blood glucose (2.4 ± 1.2 mmol/L; Figure 2B) compared to both Sham (*P* = 0.0038; Kruskal-Wallis) and IHx (*P* = 0.0171; Kruskal-Wallis) indicating that the glucoregulatory system is capable of raising blood glucose in response to a hypoxic challenge, under anesthesia.

## Discussion

The primary finding of this study was that 1 h of acute IHx is insufficient to raise blood glucose levels under pentobarbital anesthesia. In conscious animals, however, our findings agree with that of Rafacho et al. (2013), showing that 1 h of IHx raises blood glucose levels in both fasted and unfasted rats. Our findings extend those of Rafacho et al. (2013) and show that the response is not dependent on the number of hypoxic challenges in a 1 h period, the severity of the hypoxia, nor the duration of the IHx. Additionally, we also show that this response is maintained for longer periods of IHx, up to 8 h. This is important when using animal models of a human condition. IHx models are often employed to investigate mechanisms involved in the physiological or pathological processes observed in human sleep disordered breathing syndromes, such as obstructive sleep apnea (OSA). Human OSA is extraordinarily variable both between patients and within patients from night to night, but alterations in sympathetic and glucoregulatory control, associated with hypertension and diabetes are common. Animals models that mimic this variability in hypoxic challenges while producing the same outcomes are sound models.

It is well established that acute IHx in rodents under anesthesia produces long lasting sympathoexcitation (Dick et al., 2007; Xing and Pilowsky, 2010; Roy et al., 2018; Farnham et al., 2019), which can be blocked at the level of carotid bodies, the spinal cord, and brainstem (Kakall et al., 2018b; Kim et al., 2018; Farnham et al., 2019). The involvement of the sympathetic system in glucose regulation is similarly well established and involves regions within the hypothalamus (Frohman and Bernardis, 1971; Grayson et al., 2013) including the ventromedial hypothalamus (Meek et al., 2016; Shimazu and Minokoshi, 2017) and paraventricular nucleus (Sharpe et al., 2013; Menuet et al., 2014; Zhao et al., 2017), the brainstem (Verberne and Sartor, 2010; Kakall et al., 2019), the adrenal gland (Jun et al., 2014), and carotid bodies (López-Barneo, 2003). Increases in blood glucose following acute, conscious IHx can be blocked by adrenergic blockade or adrenal medullectomy (Rafacho et al., 2013; Jun et al., 2014) indicating sympathetic involvement via catecholamine release. Sympathetic involvement in glucose metabolism dysfunction was also demonstrated in humans subjected to acute IHx (Louis and Punjabi, 2009). Therefore, it seemed reasonable to assume that the sympathetically driven elevation in blood glucose would be measurable under anesthesia.

As anesthesia has varying effects on blood glucose, the choice of anesthetic was critical for the current study. Long lasting urethane anesthesia causes marked hyperglycemia (Sánchez-Pozo et al., 1988) as does the short-acting anesthetics ketamine/xylazine and isoflurane (Sano et al., 2016; Windelov et al., 2016), most likely due to sympathoadrenal stimulation and subsequent release of catecholamines from the adrenal gland (Oyama, 1973). Pentobarbital exerts its anesthetic effects by acting on inhibitory GABA receptors and hence can cause quite severe cardiorespiratory depression (Field et al., 1993), an effect described in dogs over 40 years ago (Cox and Bagshaw, 1979). Pentobarbital suppresses both sympathetic and parasympathetic arms of cardiovascular reflexes; however, is still used in studies investigating autonomic cardiovascular control (Solomon et al., 1999; Nedoboy et al., 2016; Kakall et al., 2018a) since these effects appear to be minimal if anesthetic depth is tightly controlled (Eikermann et al., 2009). Unlike the other anesthetics mentioned, pentobarbital does not raise blood glucose levels (Sano et al., 2016; Windelov et al., 2016) and is used in anesthetized studies investigating the glucoregulatory system (Korim et al., 2016), so it was chosen for this study.

Our findings following IHx combined with the previous studies indicate that pentobarbital anesthesia suppresses the autonomic reflex responses to physiological challenges, but does not abolish it, as responses can still be elicited from suprathreshold challenges. Indeed, the supraphysiological nature of the challenge is a notable feature of previously reported anesthetized experiments. To stimulate a measurable sympathoadrenal reflex, large doses of 2-DG are administered (Kakall et al., 2018a). To stimulate a similar level of catecholamine release in pentobarbital anesthetized dogs, compared with conscious dogs, a 3× greater dose of 2-DG was needed (Taborsky et al., 1984), which was also the same dose used in anesthetized rats (Kakall et al., 2018a). Our current results support this as the physiological hypoxic challenges were insufficient to raise blood glucose under pentobarbital, but a single 3 min hypoxia challenge did produce a robust response (Figure 2B). Pentobarbital was shown to prevent the elevation in glucose in response to transport in goats (Sanhouri et al., 1991), to impair the hypoglycemic effect of insulin in rats (Bailey et al., 1975) and to markedly blunt sympathoadrenal release of noradrenaline in response to hemorrhage in rats (Hamberger et al., 1984). The sympathoadrenal reflex is dependent on catecholamines and in addition to suppressing stimulated release levels, pentobarbital also suppresses resting levels (Hamberger et al., 1984; Taborsky et al., 1984). This current study did not measure catecholamines, so it is plausible that other mechanisms may contribute. Nevertheless the glucose results in the anesthetized animals are indicative of decreasing resting catecholamine levels as blood glucose levels appeared to fall in the anesthetized Sham condition but not in the conscious Sham condition, although this did not reach statistical significance.

The brain circuitry involved in driving the glucose response to acute IHx remains unknown, as does the implication of the mild increase. It is most likely that the glucose response is a sympathetically mediated stress response that serves as an important mechanism to protect the brain during acute stress by maintaining fuel supply during periods of low oxygen. There are multiple sites along the sympathetic stress axis where pentobarbital can exert its effects. Decreased levels of arterial oxygen is first sensed by the carotid bodies, located at the carotid bifurcation and are the primary oxygen sensory organs of the body. The carotid body also has a critical role in stimulating the glucose counter-regulatory response to increase blood glucose (Gao et al., 2014), but how this occurs, and whether it is direct (Gao et al., 2014) or indirect (Conde et al., 2007; O’Halloran, 2016; Thompson et al., 2016) is a matter of debate. The afferent information from the carotid bodies is conveyed to the nucleus of the solitary tract (NTS); studies showed the depressant effects of GABA or GABA agonists on ventilation when applied at the level NTS (Tabata et al., 2001), making it a potential site where pentobarbital can affect GABAergic transmission.

Hypoxia-activated NTS neurons project to multiple hypothalamic and brainstem autonomic nuclei, such as the hypothalamic paraventricular nucleus (PVN) and ventrolateral medulla (VLM). In the face of severe stress, the PVN and VLM are critical areas for mediating the hyperglycemic response (Zhao et al., 2017). Another important area of the hypothalamus for glucose homeostasis is the ventromedial hypothalamus (VMH) which projects to a wide range of sympathetic targets, including the PVN, the VLM and the NTS (Lindberg et al., 2013), which are all involved in the responses to intermittent hypoxia as described above (Mifflin et al., 2015; Shell et al., 2016; Blackburn et al., 2018; Maruyama et al., 2019). While there is no clear evidence of the neurocircuitry involved in the glycemic response to acute IHx, there is substantial knowledge of the effects of GABA on the glucoregulatory neurons in the VMH and the sympathoadrenal glucoregulatory reflex. The VMH contains glucose excited (GE) neurons that are primarily responsible for glucose utilization and regulating insulin sensitivity, as well as glucose-inhibited (GI) neurons that activate the counterregulatory reflex to raise glucose in response to falling glucose levels (Shimazu and Minokoshi, 2017). Female mice lacking glutamate receptors in the VMH have impaired insulin sensitivity and glucose regulation but without any deficit in responding to a hypoglycemia challenge (Fagan et al., 2020) suggesting that glutamate input is not the primary driver of GI neuron activation within the VMH. Inhibition of synaptic glutamate release (Tong et al., 2007) or optogenetic inhibition of neuronal firing (Meek et al., 2016) in the VMH does impair the counterregulatory reflex to hypoglycemia suggesting that the GI neurons are glutamatergic and tonically inhibited. The GI neurons that are responsible for this sympathoadrenal reflex are tonically inhibited by GABA since glucose prevents the decrease in GABA normally seen in response to hypoglycemia (Zhu et al., 2010) and antagonism of GABA (A) receptors results in a an exaggerated sympathoadrenal response to hypoglycemia (Chan et al., 2006). Elevated levels of GABA in the VMH are associated with an impaired/suppressed counterregulatory reflex (Chan et al., 2008, 2011) further highlighting the importance of GABA signaling in the brains ability to raise systemic glucose levels.

Given the abundance of GABA receptors in the central glucose- and hypoxia-sensitive areas, it is highly feasible that the GABAergic effects of pentobarbital are suppressing the glucoregulatory neurons within the central nervous system responsible for stimulating a glycemic response to acute IHx, as well as diminishing the sympathoadrenal reflex. We conclude that pentobarbital anesthesia is unsuitable for measuring the glycemic response to physiological challenges such as IHx.

## Data Availability Statement

The raw data supporting the conclusions of this article will be made available by the authors, without undue reservation.

## Ethics Statement

The animal study was reviewed and approved by the Sydney Local Area Health District Animal Care and Ethics Committee.

## Author Contributions

MF was the guarantor of this work and, as such, had full access to all the data in the study and takes responsibility for the integrity of the data, and the accuracy of the data analysis, designed the project, analyzed, and interpreted data. PN, CH, and MF conducted the experiments. MF and PN prepared the figures, wrote, revised, and edited the manuscript. All authors reviewed the manuscript.

## Conflict of Interest

The authors declare that the research was conducted in the absence of any commercial or financial relationships that could be construed as a potential conflict of interest.

## References

[B1] Bailey C. J. Atkins T. W. Matty A. J. (1975). Blood glucose and plasma insulin levels during prolonged pentobarbitone anaesthesia in the rat. *Endocrinol. Exp.* 9 177–185. 1081042

[B2] Blackburn M. B. Andrade M. A. Toney G. M. (2018). Hypothalamic PVN contributes to acute intermittent hypoxia-induced sympathetic but not phrenic long-term facilitation. *J. Appl. Physiol.* 124 1233–1243. 10.1152/japplphysiol.00743.2017 29357503 PMC6008082

[B3] Chan O. Cheng H. Herzog R. Czyzyk D. Zhu W. Wang A. (2008). Increased GABAergic tone in the ventromedial hypothalamus contributes to suppression of counterregulatory responses after antecedent hypoglycemia. *Diabetes* 57 1363–1370. 10.2337/db07-1559 18375441 PMC5518793

[B4] Chan O. Paranjape S. Czyzyk D. Horblitt A. Zhu W. Ding Y. (2011). Increased GABAergic output in the ventromedial hypothalamus contributes to impaired hypoglycemic counterregulation in diabetic rats. *Diabetes* 60 1582–1589. 10.2337/db10-1579 21411513 PMC3292334

[B5] Chan O. Zhu W. Ding Y. Mccrimmon R. J. Sherwin R. S. (2006). Blockade of GABA(A) receptors in the ventromedial hypothalamus further stimulates glucagon and sympathoadrenal but not the hypothalamo-pituitary-adrenal response to hypoglycemia. *Diabetes* 55 1080–1087. 10.2337/diabetes.55.04.06.db05-0958 16567532

[B6] Conde S. V. Obeso A. Gonzalez C. (2007). Low glucose effects on rat carotid body chemoreceptor cells’ secretory responses and action potential frequency in the carotid sinus nerve. *J. Physiol.* 585 721–730. 10.1113/jphysiol.2007.144261 17947309 PMC2375508

[B7] Cox R. H. Bagshaw R. J. (1979). Influence of anesthesia on the response to carotid hypotension in dogs. *Am. J. Physiol.* 237 H424–H432. 10.1152/ajpheart.1979.237.4.H424 495727

[B8] da Silva Paulitsch F. Zhang L. (2019). Continuous positive airway pressure for adults with obstructive sleep apnea and cardiovascular disease: a meta-analysis of randomized trials. *Sleep Med.* 54 28–34. 10.1016/j.sleep.2018.09.030 30529774

[B9] Dick T. E. Hsieh Y. H. Wang N. Prabhakar N. (2007). Acute intermittent hypoxia increases both phrenic and sympathetic nerve activities in the rat. *Exp. Physiol.* 92 87–97. 10.1113/expphysiol.2006.035758 17138622

[B10] Eikermann M. Fassbender P. Zaremba S. Jordan A. S. Rosow C. Malhotra A. (2009). Pentobarbital dose-dependently increases respiratory genioglossus muscle activity while impairing diaphragmatic function in anesthetized rats. *Anesthesiology* 110 1327–1334. 10.1097/ALN.0b013e3181a16337 19417601 PMC2727066

[B11] Fagan M. P. Ameroso D. Meng A. Rock A. Maguire J. Rios M. (2020). Essential and sex-specific effects of mGluR5 in ventromedial hypothalamus regulating estrogen signaling and glucose balance. *Proc. Natl. Acad. Sci.U.S.A.* 117 19566–19577. 10.1073/pnas.2011228117 32719118 PMC7430975

[B12] Farnham M. M. J. Tallapragada V. J. O’connor E. T. Nedoboy P. E. Dempsey B. Mohammed S. (2019). PACAP-PAC1 receptor activation is necessary for the sympathetic response to acute intermittent hypoxia. *Front. Neurosci.* 13:881. 10.3389/fnins.2019.00881 31496933 PMC6712064

[B13] Field K. J. White W. J. Lang C. M. (1993). Anaesthetic effects of chloral hydrate, pentobarbitone and urethane in adult male rats. *Lab. Anim.* 27 258–269. 10.1258/002367793780745471 8366672

[B14] Frohman L. A. Bernardis L. L. (1971). Effect of hypothalamic stimulation on plasma glucose, insulin, and glucagon levels. *Am. J. Physiol.* 221 1596–1603. 10.1152/ajplegacy.1971.221.6.1596 4941906

[B15] Fu C. Jiang L. Zhu F. Liu Z. Li W. Jiang H. (2015). Chronic intermittent hypoxia leads to insulin resistance and impaired glucose tolerance through dysregulation of adipokines in non-obese rats. *Sleep Breath.* 19 1467–1473. 10.1007/s11325-015-1144-8 25724554

[B16] Gao L. Ortega-Saenz P. Garcia-Fernandez M. Gonzalez-Rodriguez P. Caballero-Eraso C. Lopez-Barneo J. (2014). Glucose sensing by carotid body glomus cells: potential implications in disease. *Front. Physiol.* 5:398. 10.3389/fphys.2014.00398 25360117 PMC4197775

[B17] Gilmartin G. S. Lynch M. Tamisier R. Weiss J. W. (2010). Chronic intermittent hypoxia in humans during 28 nights results in blood pressure elevation and increased muscle sympathetic nerve activity. *Am. J. Physiol.* 299 H925–H931. 10.1152/ajpheart.00253.2009 20581089 PMC4116417

[B18] Gray E. L. Mckenzie D. K. Eckert D. J. (2017). Obstructive sleep apnea without obesity is common and difficult to treat: evidence for a distinct pathophysiological phenotype. *J. Clin. Sleep Med.* 13 81–88. 10.5664/jcsm.6394 27655455 PMC5181619

[B19] Grayson B. E. Seeley R. J. Sandoval D. A. (2013). Wired on sugar: the role of the CNS in the regulation of glucose homeostasis. *Nat. Rev. Neurosci.* 14 24–37. 10.1038/nrn3409 23232606 PMC4231433

[B20] Hamberger B. Bengtsson L. Jarnberg P. O. Farnebo L. O. (1984). Anesthetic agents and sympatho-adrenal response to hemorrhage in the rat. *Acta Chir. Scand. Suppl.* 520 109–113. 6594865

[B21] Heinzer R. Vat S. Marques-Vidal P. Marti-Soler H. Andries D. Tobback N. (2015). Prevalence of sleep-disordered breathing in the general population: the hypnoLaus study. *Lancet Respir. Med.* 3 310–318. 10.1016/S2213-2600(15)00043-0 25682233 PMC4404207

[B22] Hunyor I. Cook K. M. (2018). Models of intermittent hypoxia and obstructive sleep apnea: molecular pathways and their contribution to cancer. *Am. J. Physiol.* 315 R669–R687. 10.1152/ajpregu.00036.2018 29995459

[B23] Jun J. C. Shin M. K. Devera R. Yao Q. Mesarwi O. Bevans-Fonti S. (2014). Intermittent hypoxia-induced glucose intolerance is abolished by alpha-adrenergic blockade or adrenal medullectomy. *Am. J. Physiol.* 307 E1073–E1083. 10.1152/ajpendo.00373.2014 25315697 PMC4254988

[B24] Kakall Z. M. Kavurma M. M. Cohen E. M. Howe P. R. Nedoboy P. E. Pilowsky P. M. (2019). Repetitive hypoglycemia reduces activation of glucose-responsive neurons in C1 and C3 medullary brain regions to subsequent hypoglycemia. *Am. J. Physiol.* 317 E388–E398. 10.1152/ajpendo.00051.2019 31013147 PMC6732467

[B25] Kakall Z. M. Nedoboy P. E. Farnham M. M. J. Pilowsky P. M. (2018a). Activation of micro-opioid receptors in the rostral ventrolateral medulla blocks the sympathetic counterregulatory response to glucoprivation. *Am. J. Physiol.* 315 R1115–R1122. 10.1152/ajpregu.00248.2018 30499309

[B26] Kakall Z. M. Pilowsky P. M. Farnham M. M. J. (2018b). PACAP-(6-38) or kynurenate microinjections in the RVLM prevent the development of sympathetic long-term facilitation after acute intermittent hypoxia. *Am. J. Physiol.* 314 H563–H572. 10.1152/ajpheart.00596.2017 29212793

[B27] Kim S. J. Fong A. Y. Pilowsky P. M. Abbott S. B. G. (2018). Sympathoexcitation following intermittent hypoxia in rat is mediated by circulating angiotensin II acting at the carotid body and subfornical organ. *J. Physiol.* 596 3217–3232. 10.1113/JP275804 29645283 PMC6068222

[B28] Korim W. S. Llewellyn-Smith I. J. Verberne A. J. (2016). Activation of medulla-projecting perifornical neurons modulates the adrenal sympathetic response to hypoglycemia: involvement of orexin type 2 (OX2-R) receptors. *Endocrinology* 157 810–819. 10.1210/en.2015-1712 26653571

[B29] Leung R. S. Comondore V. R. Ryan C. M. Stevens D. (2012). Mechanisms of sleep-disordered breathing: causes and consequences. *Pflugers Arch.* 463 213–230. 10.1007/s00424-011-1055-x 22083643

[B30] Lindberg D. Chen P. Li C. (2013). Conditional viral tracing reveals that steroidogenic factor 1-positive neurons of the dorsomedial subdivision of the ventromedial hypothalamus project to autonomic centers of the hypothalamus and hindbrain. *J. Comp. Neurol.* 521 3167–3190. 10.1002/cne.23338 23696474

[B31] López-Barneo J. (2003). Oxygen and glucose sensing by carotid body glomus cells. *Curr. Opin. Neurobiol.* 13 493–499. 10.1016/S0959-4388(03)00093-X 12965299

[B32] Louis M. Punjabi N. M. (2009). Effects of acute intermittent hypoxia on glucose metabolism in awake healthy volunteers. *J. Appl. Physiol.* 106 1538–1544. 10.1152/japplphysiol.91523.2008 19265062 PMC2681331

[B33] Maruyama N. O. Mitchell N. C. Truong T. T. Toney G. M. (2019). Activation of the hypothalamic paraventricular nucleus by acute intermittent hypoxia: implications for sympathetic long-term facilitation neuroplasticity. *Exp. Neurol.* 314 1–8. 10.1016/j.expneurol.2018.12.011 30605624 PMC6378125

[B34] Meek T. H. Nelson J. T. Matsen M. E. Dorfman M. D. Guyenet S. J. Damian V. (2016). Functional identification of a neurocircuit regulating blood glucose. *Proc. Natl. Acad. Sci. U.S.A.* 113 E2073–E2082. 10.1073/pnas.1521160113 27001850 PMC4833243

[B35] Menuet C. Sevigny C. P. Connelly A. A. Bassi J. K. Jancovski N. Williams D. A. (2014). Catecholaminergic C3 neurons are sympathoexcitatory and involved in glucose homeostasis. *J. Neurosci.* 34 15110–15122. 10.1523/JNEUROSCI.3179-14.2014 25378174 PMC6608368

[B36] Mifflin S. Cunningham J. T. Toney G. M. (2015). Neurogenic mechanisms underlying the rapid onset of sympathetic responses to intermittent hypoxia. *J. Appl. Physiol.* 119 1441–1448. 10.1152/japplphysiol.00198.2015 25997944 PMC4683347

[B37] Narkiewicz K. Somers V. K. (1997). The sympathetic nervous system and obstructive sleep apnea: implications for hypertension. *J. Hypertens.* 15 1613–1619. 10.1097/00004872-199715120-00062 9488212

[B38] Nedoboy P. E. Mohammed S. Kapoor K. Bhandare A. M. Farnham M. M. Pilowsky P. M. (2016). pSer40 tyrosine hydroxylase immunohistochemistry identifies the anatomical location of C1 neurons in rat RVLM that are activated by hypotension. *Neuroscience* 317 162–172. 10.1016/j.neuroscience.2016.01.012 26791524

[B39] Newhouse L. P. Joyner M. J. Curry T. B. Laurenti M. C. Man C. D. Cobelli C. (2017). Three hours of intermittent hypoxia increases circulating glucose levels in healthy adults. *Physiol. Rep.* 5:e13106. 10.14814/phy2.13106 28087818 PMC5256164

[B40] O’Halloran K. D. (2016). Counter-regulatory control of homeostasis during hypoglycaemia: adrenaline hits the sweet spot in the controversy concerning carotid body glucose sensing. *J. Physiol.* 594 4091–4092. 10.1113/JP272506 27477603 PMC4967753

[B41] Oyama T. (1973). Endocrine responses to anaesthetic agents. *Br. J. Anaesth.* 45 276–281. 10.1093/bja/45.3.276 4349270

[B42] Pamidi S. Wroblewski K. Broussard J. Day A. Hanlon E. C. Abraham V. (2012). Obstructive sleep apnea in young lean men: impact on insulin sensitivity and secretion. *Diabetes Care* 35 2384–2389. 10.2337/dc12-0841 22912423 PMC3476882

[B43] Pamidi S. Tasali E. (2012). Obstructive sleep apnea and type 2 diabetes: is there a link? *Front. Neurol.* 3:126. 10.3389/fneur.2012.00126 23015803 PMC3449487

[B44] Peppard P. E. Young T. Barnet J. H. Palta M. Hagen E. W. Hla K. M. (2013). Increased prevalence of sleep-disordered breathing in adults. *Am. J. Epidemiol.* 177 1006–1014. 10.1093/aje/kws342 23589584 PMC3639722

[B45] Polak J. Shimoda L. A. Drager L. F. Undem C. Mchugh H. Polotsky V. Y. (2013). Intermittent hypoxia impairs glucose homeostasis in C57BL6/J mice: partial improvement with cessation of the exposure. *Sleep* 36 1483–1490, 1490A–1490B. 10.5665/sleep.3040 24082307 PMC3773197

[B46] Rafacho A. Goncalves-Neto L. M. Ferreira F. B. Protzek A. O. Boschero A. C. Nunes E. A. (2013). Glucose homoeostasis in rats exposed to acute intermittent hypoxia. *Acta Physiol.* 209 77–89. 10.1111/apha.12118 23692825

[B47] Rajan P. Greenberg H. (2015). Obstructive sleep apnea as a risk factor for type 2 diabetes mellitus. *Nat. Sci. Sleep* 7 113–125. 10.2147/NSS.S90835 26491377 PMC4599645

[B48] Roy A. Farnham M. M. J. Derakhshan F. Pilowsky P. M. Wilson R. J. A. (2018). Acute intermittent hypoxia with concurrent hypercapnia evokes P2X and TRPV1 receptor-dependent sensory long-term facilitation in naive carotid bodies. *J. Physiol.* 596 3149–3169. 10.1113/JP275001 29159869 PMC6068228

[B49] Sánchez-Pozo A. Alados J. C. Sánchez-Medina F. (1988). Metabolic changes induced by urethane-anesthesia in rats. *Gen. Pharmacol.* 19 281–284. 10.1016/0306-3623(88)90077-8 3350336

[B50] Sanhouri A. A. Jones R. S. Dobson H. (1991). Pentobarbitone inhibits the stress response to transport in male goats. *Br. Vet. J.* 147 42–48. 10.1016/0007-1935(91)90065-U 2018916

[B51] Sano Y. Ito S. Yoneda M. Nagasawa K. Matsuura N. Yamada Y. (2016). Effects of various types of anesthesia on hemodynamics, cardiac function, and glucose and lipid metabolism in rats. *Am. J. Physiol.* 311 H1360–H1366. 10.1152/ajpheart.00181.2016 27694213

[B52] Sharpe A. L. Calderon A. S. Andrade M. A. Cunningham J. T. Mifflin S. W. Toney G. M. (2013). Chronic intermittent hypoxia increases sympathetic control of blood pressure: role of neuronal activity in the hypothalamic paraventricular nucleus. *Am. J. Physiol.* 305 H1772–H1780. 10.1152/ajpheart.00592.2013 24097432 PMC3882549

[B53] Shell B. Faulk K. Cunningham J. T. (2016). Neural control of blood pressure in chronic intermittent hypoxia. *Curr. Hypertens. Rep.* 18:19. 10.1007/s11906-016-0627-8 26838032 PMC5080908

[B54] Shimazu T. Minokoshi Y. (2017). Systemic glucoregulation by glucose-sensing neurons in the ventromedial hypothalamic nucleus (VMH). *J. Endocr. Soc.* 1 449–459. 10.1210/js.2016-1104 29264500 PMC5686683

[B55] Solomon S. G. Llewellyn-Smith I. J. Minson J. B. Arnolda L. F. Chalmers J. P. Pilowsky P. M. (1999). Neurokinin-1 receptors and spinal cord control of blood pressure in spontaneously hypertensive rats. *Brain Res.* 815 116–120. 10.1016/S0006-8993(98)01107-X 9974130

[B56] Tabata M. Kurosawa H. Kikuchi Y. Hida W. Ogawa H. Okabe S. (2001). Role of GABA within the nucleus tractus solitarii in the hypoxic ventilatory decline of awake rats. *Am. J. Physiol.* 281 R1411–R1419. 10.1152/ajpregu.2001.281.5.R1411 11641110

[B57] Taborsky G. J. Jr. Halter J. B. Baum D. Best J. D. Porte D. Jr. (1984). Pentobarbital anesthesia suppresses basal and 2-deoxy-D-glucose-stimulated plasma catecholamines. *Am. J. Physiol.* 247 R905–R910. 10.1152/ajpregu.1984.247.5.R905 6496774

[B58] Tamisier R. Pepin J. L. Remy J. Baguet J. P. Taylor J. A. Weiss J. W. (2011). 14 nights of intermittent hypoxia elevate daytime blood pressure and sympathetic activity in healthy humans. *Eur. Respir. J.* 37 119–128. 10.1183/09031936.00204209 20525723

[B59] Thompson E. L. Ray C. J. Holmes A. P. Pye R. L. Wyatt C. N. Coney A. M. (2016). Adrenaline release evokes hyperpnoea and an increase in ventilatory CO2 sensitivity during hypoglycaemia: a role for the carotid body. *J. Physiol.* 594 4439–4452. 10.1113/JP272191 27027261 PMC4967760

[B60] Tong Q. Ye C. Mccrimmon R. J. Dhillon H. Choi B. Kramer M. D. (2007). Synaptic glutamate release by ventromedial hypothalamic neurons is part of the neurocircuitry that prevents hypoglycemia. *Cell Metab.* 5 383–393. 10.1016/j.cmet.2007.04.001 17488640 PMC1934926

[B61] Verberne A. J. Sartor D. M. (2010). Rostroventrolateral medullary neurons modulate glucose homeostasis in the rat. *Am. J. Physiol.* 299 E802–E807. 10.1152/ajpendo.00466.2010 20807841

[B62] Windelov J. A. Pedersen J. Holst J. J. (2016). Use of anesthesia dramatically alters the oral glucose tolerance and insulin secretion in C57Bl/6 mice. *Physiol. Rep.* 4:e12824. 10.14814/phy2.12824 27255361 PMC4908499

[B63] Xing T. Pilowsky P. M. (2010). Acute intermittent hypoxia in rat in vivo elicits a robust increase in tonic sympathetic nerve activity that is independent of respiratory drive. *J. Physiol.* 588 3075–3088. 10.1113/jphysiol.2010.190454 20566662 PMC2956946

[B64] Yacoub M. Youssef I. Salifu M. O. Mcfarlane S. I. (2017). Cardiovascular disease risk in obstructive sleep apnea: an update. *J. Sleep Disord. Ther.* 7:283. 10.4172/2167-0277.1000283 29644149 PMC5891150

[B65] Zhao Z. Wang L. Gao W. Hu F. Zhang J. Ren Y. (2017). A central catecholaminergic circuit controls blood glucose levels during stress. *Neuron* 95 138–152.e5. 10.1016/j.neuron.2017.05.031 28625488

[B66] Zhu W. Czyzyk D. Paranjape S. A. Zhou L. Horblitt A. Szabo G. (2010). Glucose prevents the fall in ventromedial hypothalamic GABA that is required for full activation of glucose counterregulatory responses during hypoglycemia. *Am. J. Physiol.* 298 E971–E977. 10.1152/ajpendo.00749.2009 20304763 PMC2867375

